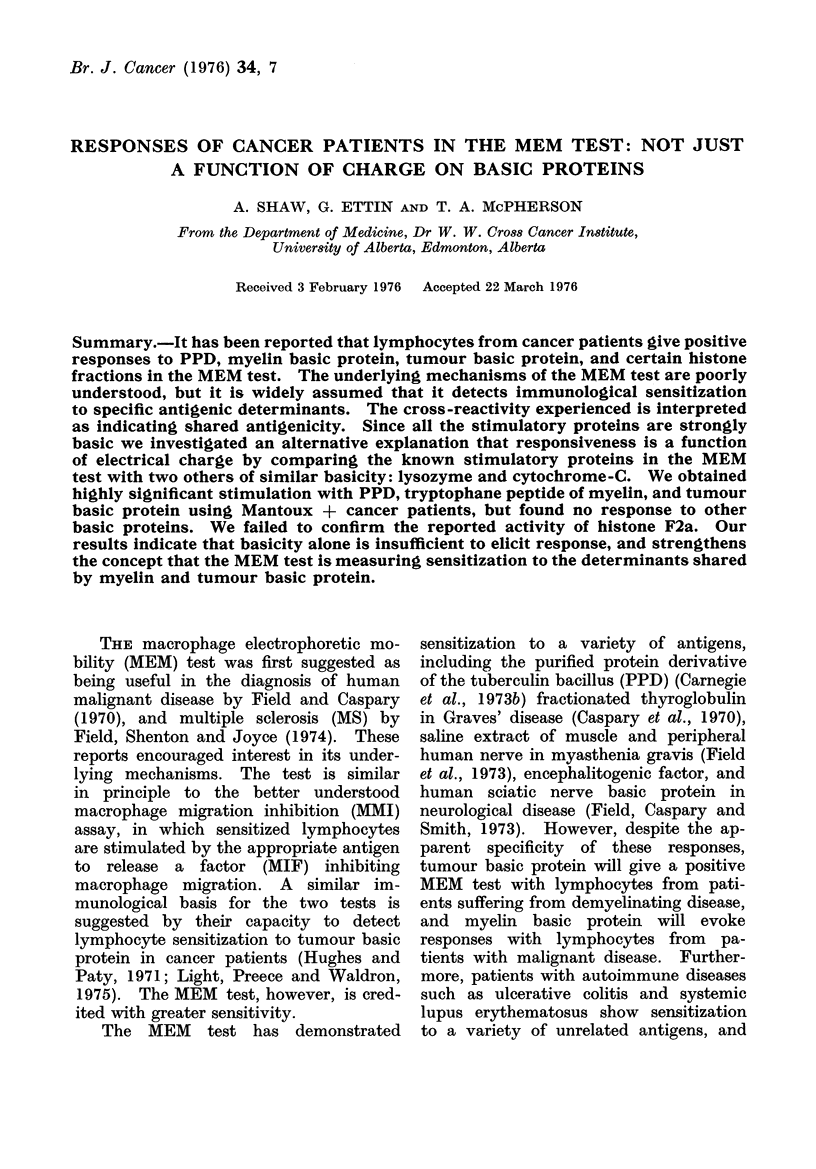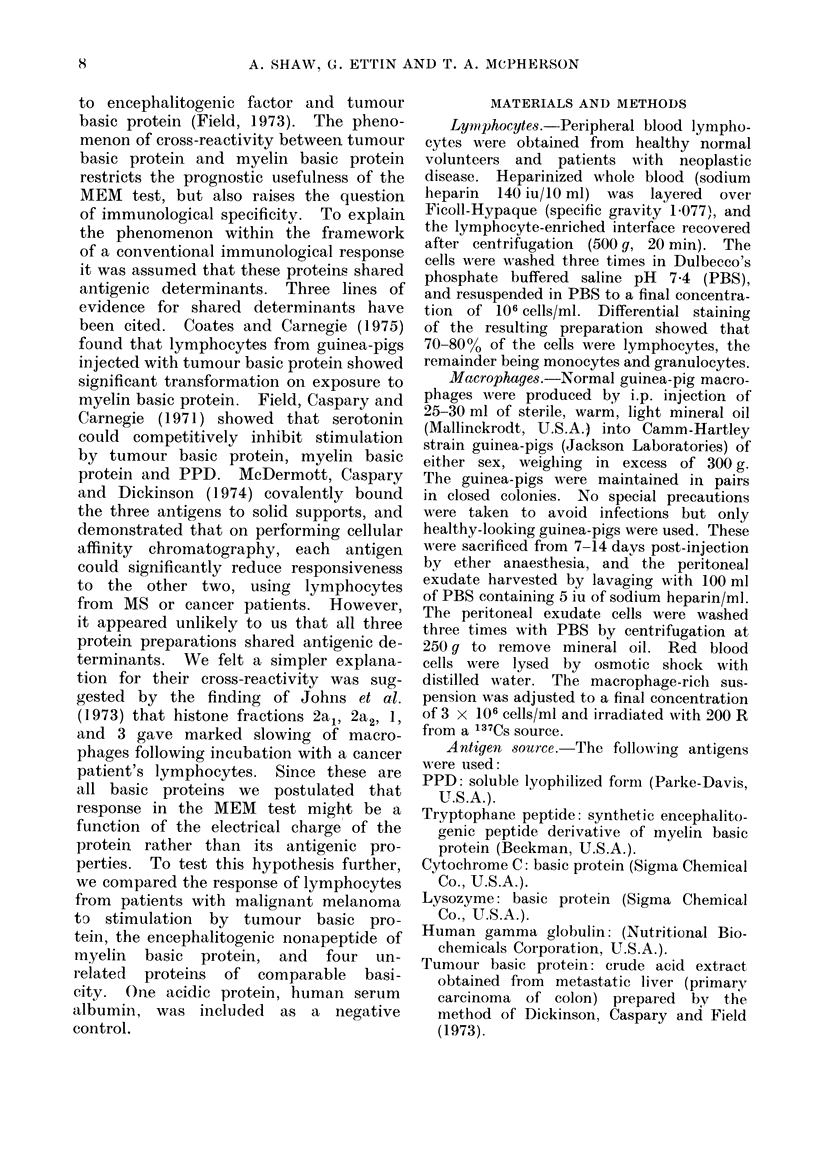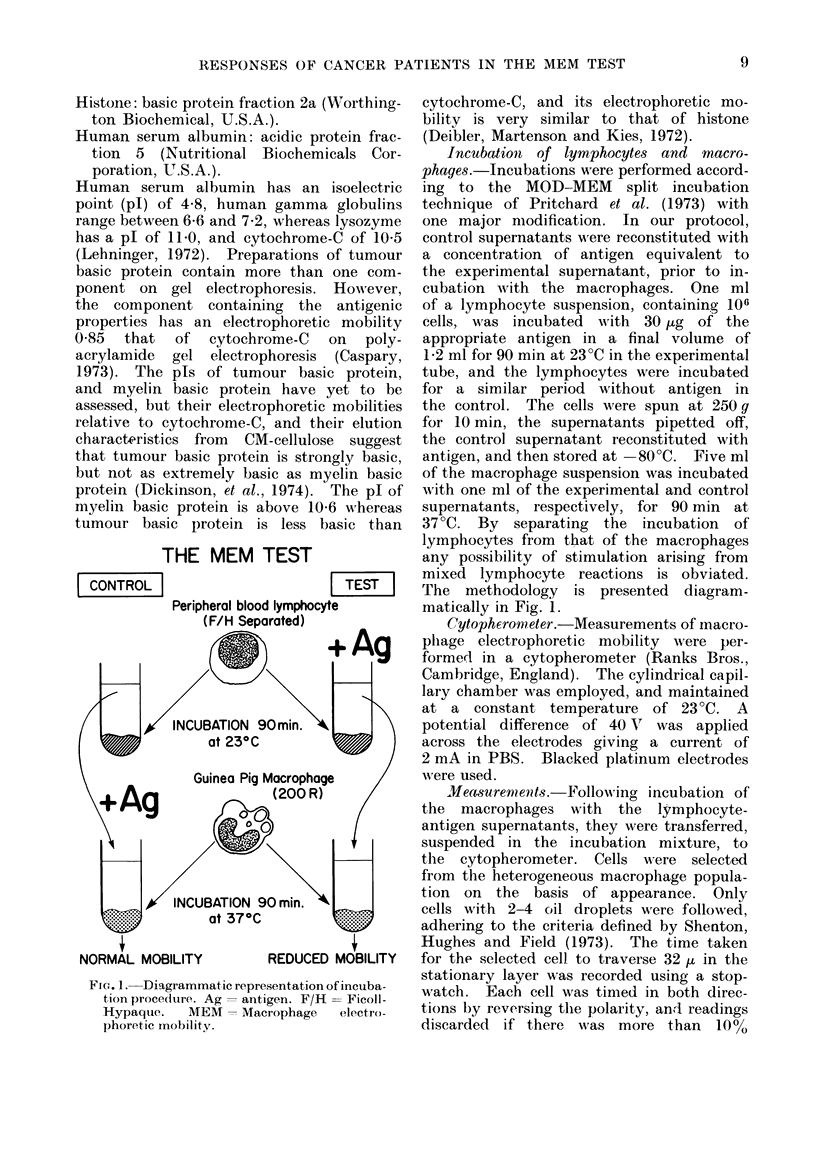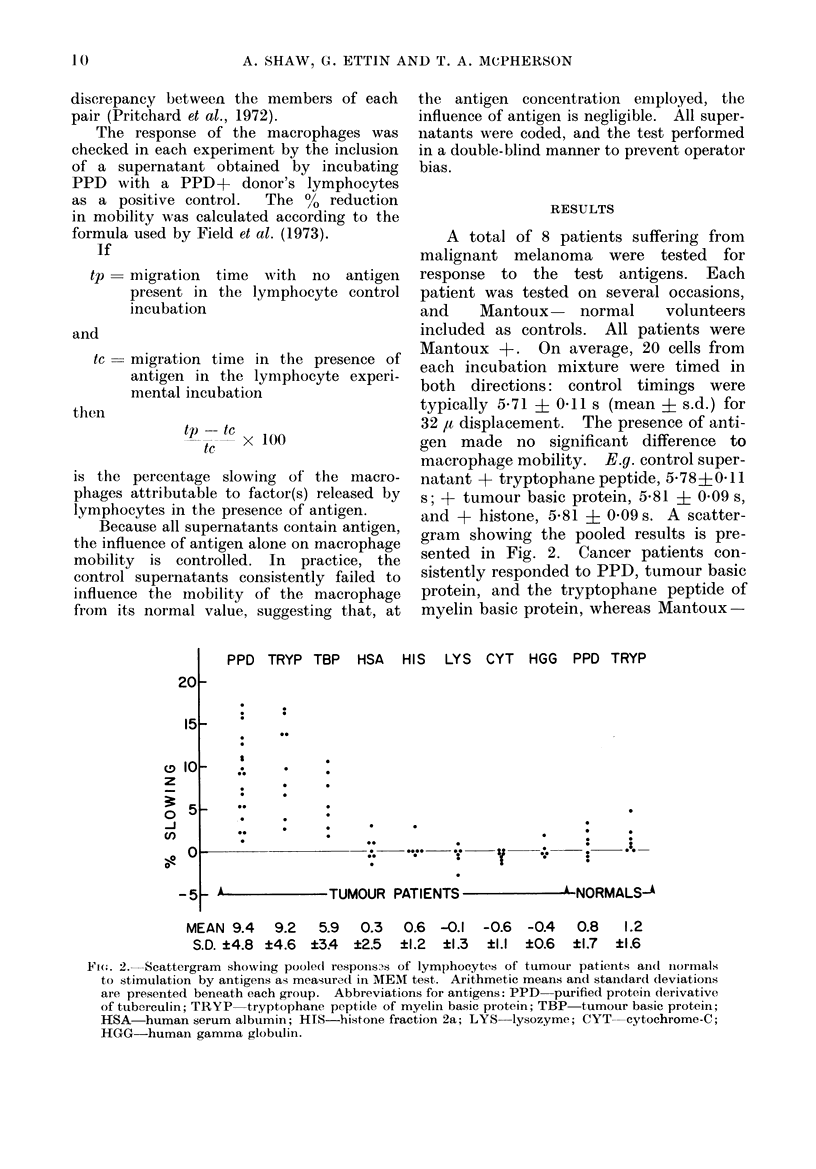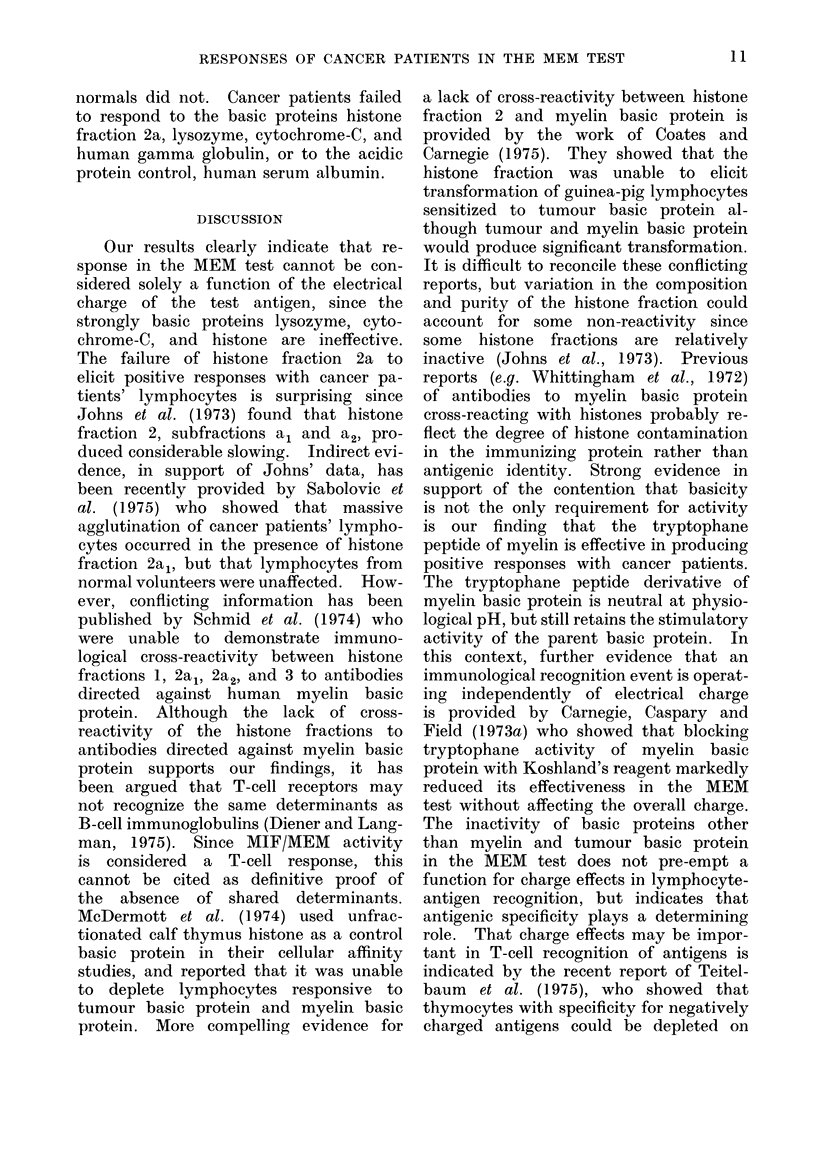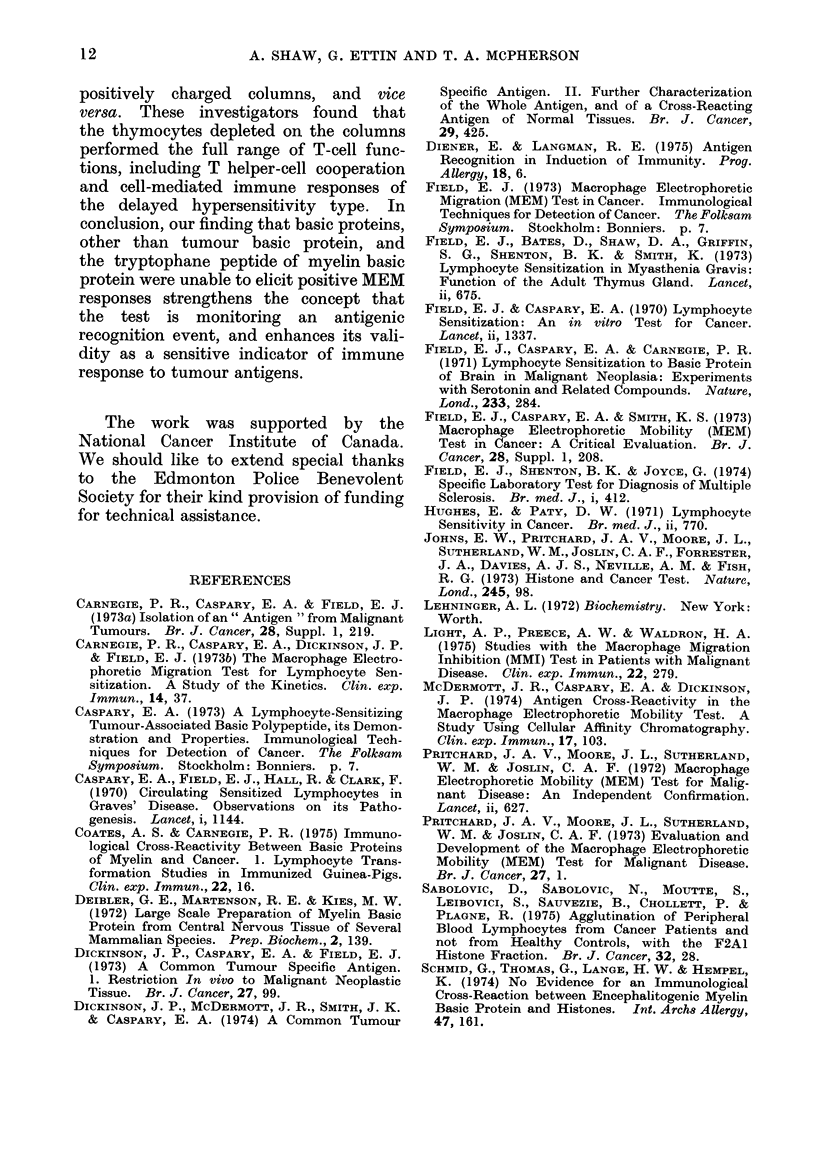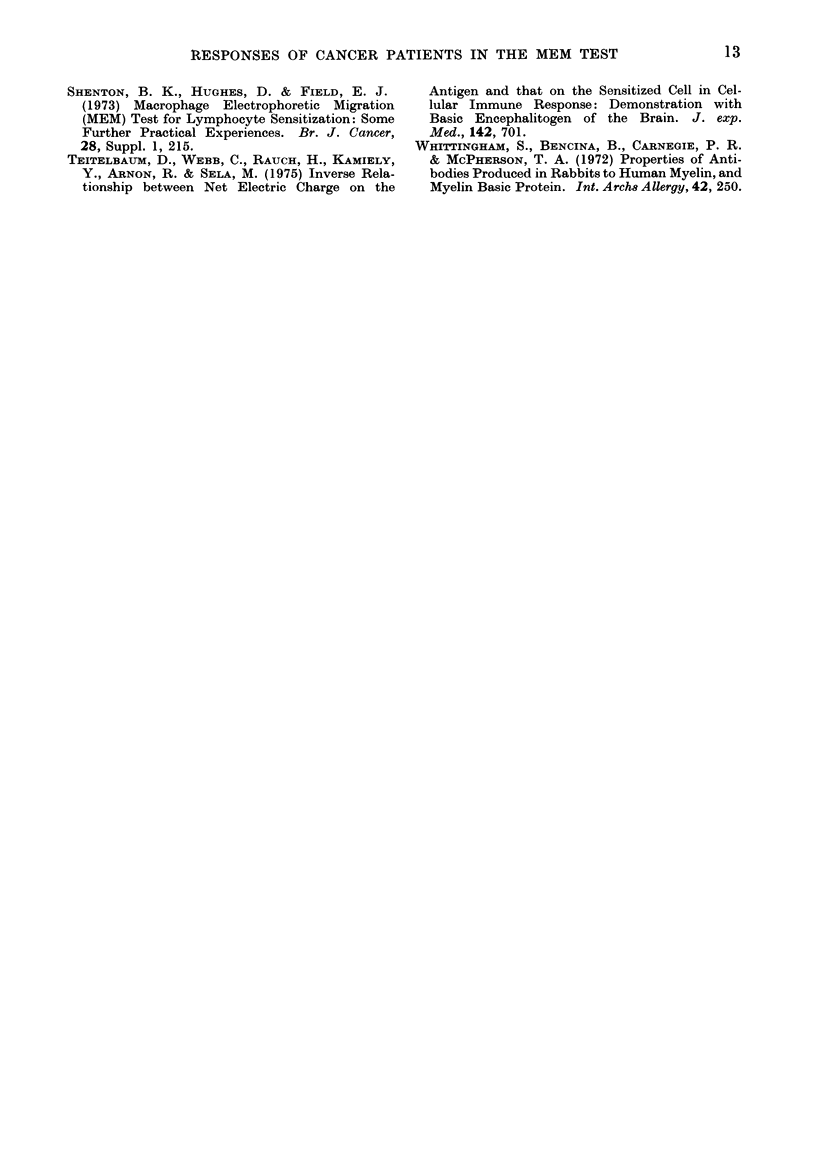# Responses of cancer patients in the MEM test: not just a function of charge on basic proteins.

**DOI:** 10.1038/bjc.1976.114

**Published:** 1976-07

**Authors:** A. Shaw, G. Ettin, T. A. McPherson

## Abstract

It has been reported that lymphocytes from cancer patients give positive responses to PPD, myelin basic protein, tumour basic protein, and certain histone fractions in the MEM test. The underlying mechanisms of the MEM test are poorly understood, but it is widely assumed that it detects immunological sensitization to specific antigenic determinants. The cross-reactivity experienced is interpreted as indicating shared antigenicity. Since all the stimulatory proteins are strongly basic we investigated an alternative explanation that responsiveness is a function of electrical charge by comparing the known stimulatory proteins in the MEM test with two others of similar basicity: lysozyme and cytochrome-C. We obtained highly significant stimulation with PPD, tryptophane peptide of myelin, and tumour basic protein using Mantoux + cancer patients, but found no response to other basic proteins. We failed to confirm the reported activity of histone F2a. Our results indicate that basicity alone is insufficient to elicit response, and strengthens the concept that the MEM test is measuring sensitization to the determinants shared by myelin and tumour basic protein.


*Br. J. Cancer* (1976) **34**, 7

### RESPONSES OF CANCER PATIENTS IN THE MEM TEST: NOT JUST A FUNCTION OF CHARGE ON BASIC PROTEINS

A. SHAW, G. ETTIN AND T. A. McPHERSON

*From the Department of Medicine, Dr W. W. Cross Cancer Institute, University of Alberta, Edmonton, Alberta*

Received 3 February 1976 Accepted 22 March 1976

**Summary.—It has been reported that lymphocytes from cancer patients give positive responses to PPD, myelin basic protein, tumour basic protein, and certain histone fractions in the MEM test. The underlying mechanisms of the MEM test are poorly understood, but it is widely assumed that it detects immunological sensitization to specific antigenic determinants. The cross-reactivity experienced is interpreted as indicating shared antigenicity. Since all the stimulatory proteins are strongly basic we investigated an alternative explanation that responsiveness is a function of electrical charge by comparing the known stimulatory proteins in the MEM test with two others of similar basicity: lysozyme and cytochrome-C. We obtained highly significant stimulation with PPD, tryptophane peptide of myelin, and tumour basic protein using Mantoux + cancer patients, but found no response to other basic proteins. We failed to confirm the reported activity of histone F2a. Our results indicate that basicity alone is insufficient to elicit response, and strengthens the concept that the MEM test is measuring sensitization to the determinants shared by myelin and tumour basic protein.**

THE macrophage electrophoretic mobility (MEM) test was first suggested as being useful in the diagnosis of human malignant disease by Field and Caspary (1970), and multiple sclerosis (MS) by Field, Shenton and Joyce (1974). These reports encouraged interest in its underlying mechanisms. The test is similar in principle to the better understood macrophage migration inhibition (MMI) assay, in which sensitized lymphocytes are stimulated by the appropriate antigen to release a factor (MIF) inhibiting macrophage migration. A similar immunological basis for the two tests is suggested by their capacity to detect lymphocyte sensitization to tumour basic protein in cancer patients (Hughes and Paty, 1971; Light, Preece and Waldron, 1975). The MEM test, however, is credited with greater sensitivity.

The MEM test has demonstrated sensitization to a variety of antigens, including the purified protein derivative of the tuberculin bacillus (PPD) (Carnegie *et al.*, 1973*b*) fractionated thyroglobulin in Graves' disease (Caspary *et al.*, 1970), saline extract of muscle and peripheral human nerve in myasthenia gravis (Field *et al.*, 1973), encephalitogenic factor, and human sciatic nerve basic protein in neurological disease (Field, Caspary and Smith, 1973). However, despite the apparent specificity of these responses, tumour basic protein will give a positive MEM test with lymphocytes from patients suffering from demyelinating disease, and myelin basic protein will evoke responses with lymphocytes from patients with malignant disease. Furthermore, patients with autoimmune diseases such as ulcerative colitis and systemic lupus erythematosus show sensitization to a variety of unrelated antigens, and to encephalitogenic factor and tumour basic protein (Field, 1973). The phenomenon of cross-reactivity between tumour basic protein and myelin basic protein restricts the prognostic usefulness of the MEM test, but also raises the question of immunological specificity. To explain the phenomenon within the framework of a conventional immunological response it was assumed that these proteins shared antigenic determinants. Three lines of evidence for shared determinants have been cited. Coates and Carnegie (1975) found that lymphocytes from guinea-pigs injected with tumour basic protein showed significant transformation on exposure to myelin basic protein. Field, Caspary and Carnegie (1971) showed that serotonin could competitively inhibit stimulation by tumour basic protein, myelin basic protein and PPD. McDermott, Caspary and Dickinson (1974) covalently bound the three antigens to solid supports, and demonstrated that on performing cellular affinity chromatography, each antigen could significantly reduce responsiveness to the other two, using lymphocytes from MS or cancer patients. However, it appeared unlikely to us that all three protein preparations shared antigenic determinants. We felt a simpler explanation for their cross-reactivity was suggested by the finding of Johns *et al.* (1973) that histone fractions $2a_1$, $2a_2$, 1, and 3 gave marked slowing of macrophages following incubation with a cancer patient's lymphocytes. Since these are all basic proteins we postulated that response in the MEM test might be a function of the electrical charge of the protein rather than its antigenic properties. To test this hypothesis further, we compared the response of lymphocytes from patients with malignant melanoma to stimulation by tumour basic protein, the encephalitogenic nonapeptide of myelin basic protein, and four unrelated proteins of comparable basicity. One acidic protein, human serum albumin, was included as a negative control.

### MATERIALS AND METHODS

*Lymphocytes.*—Peripheral blood lymphocytes were obtained from healthy normal volunteers and patients with neoplastic disease. Heparinized whole blood (sodium heparin 140 iu/10 ml) was layered over Ficoll-Hypaque (specific gravity 1·077), and the lymphocyte-enriched interface recovered after centrifugation (500 *g*, 20 min). The cells were washed three times in Dulbecco's phosphate buffered saline pH 7·4 (PBS), and resuspended in PBS to a final concentration of $10^6$ cells/ml. Differential staining of the resulting preparation showed that 70–80% of the cells were lymphocytes, the remainder being monocytes and granulocytes.

*Macrophages.*—Normal guinea-pig macrophages were produced by i.p. injection of 25–30 ml of sterile, warm, light mineral oil (Mallinckrodt, U.S.A.) into Camm-Hartley strain guinea-pigs (Jackson Laboratories) of either sex, weighing in excess of 300 g. The guinea-pigs were maintained in pairs in closed colonies. No special precautions were taken to avoid infections but only healthy-looking guinea-pigs were used. These were sacrificed from 7–14 days post-injection by ether anaesthesia, and the peritoneal exudate harvested by lavaging with 100 ml of PBS containing 5 iu of sodium heparin/ml. The peritoneal exudate cells were washed three times with PBS by centrifugation at 250 *g* to remove mineral oil. Red blood cells were lysed by osmotic shock with distilled water. The macrophage-rich suspension was adjusted to a final concentration of $3 \times 10^6$ cells/ml and irradiated with 200 R from a $^{137}$Cs source.

*Antigen source.*—The following antigens were used:

PPD: soluble lyophilized form (Parke-Davis, U.S.A.).

Tryptophane peptide: synthetic encephalitogenic peptide derivative of myelin basic protein (Beckman, U.S.A.).

Cytochrome C: basic protein (Sigma Chemical Co., U.S.A.).

Lysozyme: basic protein (Sigma Chemical Co., U.S.A.).

Human gamma globulin: (Nutritional Biochemicals Corporation, U.S.A.).

Tumour basic protein: crude acid extract obtained from metastatic liver (primary carcinoma of colon) prepared by the method of Dickinson, Caspary and Field (1973).

Histone: basic protein fraction 2a (Worthington Biochemical, U.S.A.).
Human serum albumin: acidic protein fraction 5 (Nutritional Biochemicals Corporation, U.S.A.).

Human serum albumin has an isoelectric point (pI) of 4·8, human gamma globulins range between 6·6 and 7·2, whereas lysozyme has a pI of 11·0, and cytochrome-C of 10·5 (Lehninger, 1972). Preparations of tumour basic protein contain more than one component on gel electrophoresis. However, the component containing the antigenic properties has an electrophoretic mobility 0·85 that of cytochrome-C on polyacrylamide gel electrophoresis (Caspary, 1973). The pIs of tumour basic protein, and myelin basic protein have yet to be assessed, but their electrophoretic mobilities relative to cytochrome-C, and their elution characteristics from CM-cellulose suggest that tumour basic protein is strongly basic, but not as extremely basic as myelin basic protein (Dickinson, *et al.*, 1974). The pI of myelin basic protein is above 10·6 whereas tumour basic protein is less basic than cytochrome-C, and its electrophoretic mobility is very similar to that of histone (Deibler, Martenson and Kies, 1972).

Fig. 1.—Diagrammatic representation of incubation procedure. Ag = antigen. F/H = Ficoll-Hypaque. MEM = Macrophage electrophoretic mobility.

*Incubation of lymphocytes and macrophages.*—Incubations were performed according to the MOD–MEM split incubation technique of Pritchard *et al.* (1973) with one major modification. In our protocol, control supernatants were reconstituted with a concentration of antigen equivalent to the experimental supernatant, prior to incubation with the macrophages. One ml of a lymphocyte suspension, containing $10^6$ cells, was incubated with 30 $\mu$g of the appropriate antigen in a final volume of 1·2 ml for 90 min at 23°C in the experimental tube, and the lymphocytes were incubated for a similar period without antigen in the control. The cells were spun at 250 *g* for 10 min, the supernatants pipetted off, the control supernatant reconstituted with antigen, and then stored at −80°C. Five ml of the macrophage suspension was incubated with one ml of the experimental and control supernatants, respectively, for 90 min at 37°C. By separating the incubation of lymphocytes from that of the macrophages any possibility of stimulation arising from mixed lymphocyte reactions is obviated. The methodology is presented diagrammatically in Fig. 1.

*Cytopherometer.*—Measurements of macrophage electrophoretic mobility were performed in a cytopherometer (Ranks Bros., Cambridge, England). The cylindrical capillary chamber was employed, and maintained at a constant temperature of 23°C. A potential difference of 40 V was applied across the electrodes giving a current of 2 mA in PBS. Blacked platinum electrodes were used.

*Measurements.*—Following incubation of the macrophages with the lymphocyte-antigen supernatants, they were transferred, suspended in the incubation mixture, to the cytopherometer. Cells were selected from the heterogeneous macrophage population on the basis of appearance. Only cells with 2–4 oil droplets were followed, adhering to the criteria defined by Shenton, Hughes and Field (1973). The time taken for the selected cell to traverse 32 $\mu$ in the stationary layer was recorded using a stopwatch. Each cell was timed in both directions by reversing the polarity, and readings discarded if there was more than 10%

discrepancy between the members of each pair (Pritchard *et al.*, 1972).

The response of the macrophages was checked in each experiment by the inclusion of a supernatant obtained by incubating PPD with a PPD+ donor's lymphocytes as a positive control. The % reduction in mobility was calculated according to the formula used by Field *et al.* (1973).

If

$tp$ = migration time with no antigen present in the lymphocyte control incubation and

$tc$ = migration time in the presence of antigen in the lymphocyte experimental incubation then

$$\frac{tp - tc}{tc} \times 100$$

is the percentage slowing of the macrophages attributable to factor(s) released by lymphocytes in the presence of antigen.

Because all supernatants contain antigen, the influence of antigen alone on macrophage mobility is controlled. In practice, the control supernatants consistently failed to influence the mobility of the macrophage from its normal value, suggesting that, at the antigen concentration employed, the influence of antigen is negligible. All supernatants were coded, and the test performed in a double-blind manner to prevent operator bias.

### RESULTS

A total of 8 patients suffering from malignant melanoma were tested for response to the test antigens. Each patient was tested on several occasions, and Mantoux— normal volunteers included as controls. All patients were Mantoux +. On average, 20 cells from each incubation mixture were timed in both directions: control timings were typically 5·71 ± 0·11 s (mean ± s.d.) for 32 $\mu$ displacement. The presence of antigen made no significant difference to macrophage mobility. *E.g.* control supernatant + tryptophane peptide, 5·78±0·11 s; + tumour basic protein, 5·81 ± 0·09 s, and + histone, 5·81 ± 0·09 s. A scattergram showing the pooled results is presented in Fig. 2. Cancer patients consistently responded to PPD, tumour basic protein, and the tryptophane peptide of myelin basic protein, whereas Mantoux—

FIG. 2.—Scattergram showing pooled responses of lymphocytes of tumour patients and normals to stimulation by antigens as measured in MEM test. Arithmetic means and standard deviations are presented beneath each group. Abbreviations for antigens: PPD—purified protein derivative of tuberculin; TRYP—tryptophane peptide of myelin basic protein; TBP—tumour basic protein; HSA—human serum albumin; HIS—histone fraction 2a; LYS—lysozyme; CYT—cytochrome-C; HGG—human gamma globulin.

normals did not. Cancer patients failed to respond to the basic proteins histone fraction 2a, lysozyme, cytochrome-C, and human gamma globulin, or to the acidic protein control, human serum albumin.

### DISCUSSION

Our results clearly indicate that response in the MEM test cannot be considered solely a function of the electrical charge of the test antigen, since the strongly basic proteins lysozyme, cytochrome-C, and histone are ineffective. The failure of histone fraction 2a to elicit positive responses with cancer patients' lymphocytes is surprising since Johns *et al.* (1973) found that histone fraction 2, subfractions $a_1$ and $a_2$, produced considerable slowing. Indirect evidence, in support of Johns' data, has been recently provided by Sabolovic *et al.* (1975) who showed that massive agglutination of cancer patients' lymphocytes occurred in the presence of histone fraction $2a_1$, but that lymphocytes from normal volunteers were unaffected. However, conflicting information has been published by Schmid *et al.* (1974) who were unable to demonstrate immunological cross-reactivity between histone fractions 1, $2a_1$, $2a_2$, and 3 to antibodies directed against human myelin basic protein. Although the lack of cross-reactivity of the histone fractions to antibodies directed against myelin basic protein supports our findings, it has been argued that T-cell receptors may not recognize the same determinants as B-cell immunoglobulins (Diener and Langman, 1975). Since MIF/MEM activity is considered a T-cell response, this cannot be cited as definitive proof of the absence of shared determinants. McDermott *et al.* (1974) used unfractionated calf thymus histone as a control basic protein in their cellular affinity studies, and reported that it was unable to deplete lymphocytes responsive to tumour basic protein and myelin basic protein. More compelling evidence for a lack of cross-reactivity between histone fraction 2 and myelin basic protein is provided by the work of Coates and Carnegie (1975). They showed that the histone fraction was unable to elicit transformation of guinea-pig lymphocytes sensitized to tumour basic protein although tumour and myelin basic protein would produce significant transformation. It is difficult to reconcile these conflicting reports, but variation in the composition and purity of the histone fraction could account for some non-reactivity since some histone fractions are relatively inactive (Johns *et al.*, 1973). Previous reports (*e.g.* Whittingham *et al.*, 1972) of antibodies to myelin basic protein cross-reacting with histones probably reflect the degree of histone contamination in the immunizing protein rather than antigenic identity. Strong evidence in support of the contention that basicity is not the only requirement for activity is our finding that the tryptophane peptide of myelin is effective in producing positive responses with cancer patients. The tryptophane peptide derivative of myelin basic protein is neutral at physiological pH, but still retains the stimulatory activity of the parent basic protein. In this context, further evidence that an immunological recognition event is operating independently of electrical charge is provided by Carnegie, Caspary and Field (1973*a*) who showed that blocking tryptophane activity of myelin basic protein with Koshland's reagent markedly reduced its effectiveness in the MEM test without affecting the overall charge. The inactivity of basic proteins other than myelin and tumour basic protein in the MEM test does not pre-empt a function for charge effects in lymphocyte-antigen recognition, but indicates that antigenic specificity plays a determining role. That charge effects may be important in T-cell recognition of antigens is indicated by the recent report of Teitelbaum *et al.* (1975), who showed that thymocytes with specificity for negatively charged antigens could be depleted on positively charged columns, and *vice versa*. These investigators found that the thymocytes depleted on the columns performed the full range of T-cell functions, including T helper-cell cooperation and cell-mediated immune responses of the delayed hypersensitivity type. In conclusion, our finding that basic proteins, other than tumour basic protein, and the tryptophane peptide of myelin basic protein were unable to elicit positive MEM responses strengthens the concept that the test is monitoring an antigenic recognition event, and enhances its validity as a sensitive indicator of immune response to tumour antigens.

The work was supported by the National Cancer Institute of Canada. We should like to extend special thanks to the Edmonton Police Benevolent Society for their kind provision of funding for technical assistance.

REFERENCES

CARNEGIE, P. R., CASPARY, E. A. & FIELD, E. J. (1973*a*) Isolation of an " Antigen " from Malignant Tumours. *Br. J. Cancer*, **28**, Suppl. 1, 219.

CARNEGIE, P. R., CASPARY, E. A., DICKINSON, J. P. & FIELD, E. J. (1973*b*) The Macrophage Electrophoretic Migration Test for Lymphocyte Sensitization. A Study of the Kinetics. *Clin. exp. Immun.*, **14**, 37.

CASPARY, E. A. (1973) A Lymphocyte-Sensitizing Tumour-Associated Basic Polypeptide, its Demonstration and Properties. Immunological Techniques for Detection of Cancer. *The Folksam Symposium*. Stockholm: Bonniers. p. 7.

CASPARY, E. A., FIELD, E. J., HALL, R. & CLARK, F. (1970) Circulating Sensitized Lymphocytes in Graves' Disease. Observations on its Pathogenesis. *Lancet*, i, 1144.

COATES, A. S. & CARNEGIE, P. R. (1975) Immunological Cross-Reactivity Between Basic Proteins of Myelin and Cancer. 1. Lymphocyte Transformation Studies in Immunized Guinea-Pigs. *Clin. exp. Immun.*, **22**, 16.

DEIBLER, G. E., MARTENSON, R. E. & KIES, M. W. (1972) Large Scale Preparation of Myelin Basic Protein from Central Nervous Tissue of Several Mammalian Species. *Prep. Biochem.*, **2**, 139.

DICKINSON, J. P., CASPARY, E. A. & FIELD, E. J. (1973) A Common Tumour Specific Antigen. 1. Restriction *In vivo* to Malignant Neoplastic Tissue. *Br. J. Cancer*, **27**, 99.

DICKINSON, J. P., McDERMOTT, J. R., SMITH, J. K. & CASPARY, E. A. (1974) A Common Tumour Specific Antigen. II. Further Characterization of the Whole Antigen, and of a Cross-Reacting Antigen of Normal Tissues. *Br. J. Cancer*, **29**, 425.

DIENER, E. & LANGMAN, R. E. (1975) Antigen Recognition in Induction of Immunity. *Prog. Allergy*, **18**, 6.

FIELD, E. J. (1973) Macrophage Electrophoretic Migration (MEM) Test in Cancer. Immunological Techniques for Detection of Cancer. *The Folksam Symposium*. Stockholm: Bonniers. p. 7.

FIELD, E. J., BATES, D., SHAW, D. A., GRIFFIN, S. G., SHENTON, B. K. & SMITH, K. (1973) Lymphocyte Sensitization in Myasthenia Gravis: Function of the Adult Thymus Gland. *Lancet*, ii, 675.

FIELD, E. J. & CASPARY, E. A. (1970) Lymphocyte Sensitization: An *in vitro* Test for Cancer. *Lancet*, ii, 1337.

FIELD, E. J., CASPARY, E. A. & CARNEGIE, P. R. (1971) Lymphocyte Sensitization to Basic Protein of Brain in Malignant Neoplasia: Experiments with Serotonin and Related Compounds. *Nature, Lond.*, **233**, 284.

FIELD, E. J., CASPARY, E. A. & SMITH, K. S. (1973) Macrophage Electrophoretic Mobility (MEM) Test in Cancer: A Critical Evaluation. *Br. J. Cancer*, **28**, Suppl. 1, 208.

FIELD, E. J., SHENTON, B. K. & JOYCE, G. (1974) Specific Laboratory Test for Diagnosis of Multiple Sclerosis. *Br. med. J.*, i, 412.

HUGHES, E. & PATY, D. W. (1971) Lymphocyte Sensitivity in Cancer. *Br. med. J.*, ii, 770.

JOHNS, E. W., PRITCHARD, J. A. V., MOORE, J. L., SUTHERLAND, W. M., JOSLIN, C. A. F., FORRESTER, J. A., DAVIES, A. J. S., NEVILLE, A. M. & FISH, R. G. (1973) Histone and Cancer Test. *Nature, Lond.*, **245**, 98.

LEHNINGER, A. L. (1972) *Biochemistry*. New York: Worth.

LIGHT, A. P., PREECE, A. W. & WALDRON, H. A. (1975) Studies with the Macrophage Migration Inhibition (MMI) Test in Patients with Malignant Disease. *Clin. exp. Immun.*, **22**, 279.

McDERMOTT, J. R., CASPARY, E. A. & DICKINSON, J. P. (1974) Antigen Cross-Reactivity in the Macrophage Electrophoretic Mobility Test. A Study Using Cellular Affinity Chromatography. *Clin. exp. Immun.*, **17**, 103.

PRITCHARD, J. A. V., MOORE, J. L., SUTHERLAND, W. M. & JOSLIN, C. A. F. (1972) Macrophage Electrophoretic Mobility (MEM) Test for Malignant Disease: An Independent Confirmation. *Lancet*, ii, 627.

PRITCHARD, J. A. V., MOORE, J. L., SUTHERLAND, W. M. & JOSLIN, C. A. F. (1973) Evaluation and Development of the Macrophage Electrophoretic Mobility (MEM) Test for Malignant Disease. *Br. J. Cancer*, **27**, 1.

SABOLOVIC, D., SABOLOVIC, N., MOUTTE, S., LEIBOVICI, S., SAUVEZIE, B., CHOLLETT, P. & PLAGNE, R. (1975) Agglutination of Peripheral Blood Lymphocytes from Cancer Patients and not from Healthy Controls, with the F2A1 Histone Fraction. *Br. J. Cancer*, **32**, 28.

SCHMID, G., THOMAS, G., LANGE, H. W. & HEMPEL, K. (1974) No Evidence for an Immunological Cross-Reaction between Encephalitogenic Myelin Basic Protein and Histones. *Int. Archs Allergy*, **47**, 161.

SHENTON, B. K., HUGHES, D. & FIELD, E. J. (1973) Macrophage Electrophoretic Migration (MEM) Test for Lymphocyte Sensitization: Some Further Practical Experiences. *Br. J. Cancer*, **28**, Suppl. 1, 215.

TEITELBAUM, D., WEBB, C., RAUCH, H., KAMIELY, Y., ARNON, R. & SELA, M. (1975) Inverse Relationship between Net Electric Charge on the Antigen and that on the Sensitized Cell in Cellular Immune Response: Demonstration with Basic Encephalitogen of the Brain. *J. exp. Med.*, **142**, 701.

WHITTINGHAM, S., BENCINA, B., CARNEGIE, P. R. & MCPHERSON, T. A. (1972) Properties of Antibodies Produced in Rabbits to Human Myelin, and Myelin Basic Protein. *Int. Archs Allergy*, **42**, 250.